# Increased expression of ZEB1-AS1 correlates with higher histopathological grade and promotes tumorigenesis in bladder cancer

**DOI:** 10.18632/oncotarget.15527

**Published:** 2017-02-20

**Authors:** Junhao Lin, Yonghao Zhan, Yuchen Liu, Zhicong Chen, Jianbo Liang, Wei Li, Anbang He, Liqun Zhou, Hongbin Mei, Feng Wang, Weiren Huang

**Affiliations:** ^1^ Department of Urology, State Engineering Laboratory of Medical Key Technologies Application of Synthetic Biology, Key Laboratory of Medical Reprogramming Technology, Shenzhen Second People's Hospital, The First Affiliated Hospital of Shenzhen University, Shenzhen 518035, Guangdong, China; ^2^ Department of Urology, Peking University First Hospital, Institute of Urology, Peking University, National Urological Cancer Centre, 100034, Beijing, China; ^3^ Department of Urology, The People's Hospital of Guangxi Zhuang Autonomous Region, Nanning 530021, Guangxi, China

**Keywords:** long non-coding RNA, ZEB1-AS1, tumorigenesis, histopathological grade, bladder cancer

## Abstract

Bladder cancer is one of the most common urinary cancers worldwide. Emerging studies indicated that long non-coding RNAs (lncRNAs) play crucial roles in cancer biology. In this study, we found that a novel lncRNA Zinc finger E-box-binding homeebox1 (ZEB1) antisense RNA (ZEB1-AS1) was overexpressed in bladder cancer tissues compared to paired noncancerous tissues. Moreover, the expression of ZEB1-AS1 was positive correlated with higher histological grade and TNM stage in bladder cancer. Furthermore, Loss-of-function experiments showed that down-regulation of ZEB1-AS1 not only can suppress cell growth but also can inhibit migration and induce apoptosis in bladder cancer cell lines 5637 and SW780. In conclusion, these findings indicated that ZEB1-AS1 plays regulatory roles in bladder cancer and it may become a novel molecular biomarker of prognosis and therapy in bladder cancer.

## INTRODUCTION

Bladder cancer is a frequent cause of cancer-related death wordwide [1]. Surgery and chemotherapy remain the curative treatment for bladder cancer, but the survival rate is far from satisfactory [2, 3]. Although numerous genes correlated with tumorigenesis and tumor metastasis were found, the molecular mechanism is still unknown [4]. Therefore, revealing the molecular mechanism may provide an effective therapy or prognostic prediction for bladder cancer.

Long non-coding RNAs (lncRNAs), longer than 200 nucleotides in length, are one of the two major classes among non-coding RNAs [5]. With the development of gene-wide studies, the lncRNAs have been shown to be associated with the development of human diseases, especially in cancers [6–8]. Accumulating evidence suggested that lncRNAs expression was elevated or descending in cancer tissues and specific lnRNAs were related with different kinds of cancer [9–11]. Examples include HOTAIR in breast cancer [10, 11], ANRIL in prostate cancer [12] and MALAT1 in bladder cancer and lung cancer [13, 14].

Zinc finger E-box-binding homeobox1 (ZEB1), as a transcription factor, is a repressor of E-cadherin [15, 16]. Recent studies showed that a novel long non-coding RNA ZEB1 antisense1 (ZEB1-AS1) was an antisense transcript which originated from the promoters of ZEB1. Moreover, studies also showed that ZEB1-AS1 could positively regulate the ZEB1 expression and promote cell growth and metastasis in Hepatocellular Carcinoma (HCC) [17]. However, whether ZEB-AS1 participates in carcinogenesis and progression of bladder cancer is still unknown and needs to be studied.

In this study, we found that ZEB1-AS1 expression was upregulated in bladder cancer tissues compared with paired non-tumor tissues and correlated with higher histological grade and TNM stage in a cohort of 55 patients with bladder cancer. Furthermore, the results of loss-of-function experiments indicated that ZEB1-AS1 acts as an oncogene and promotes malignant phenotypes of bladder cancer cells.

## RESULTS

### ZEB1-AS1 is over-expressed in bladder cancer tissues, cells and it is correlated with clinicopathological features

**Figure 1:**
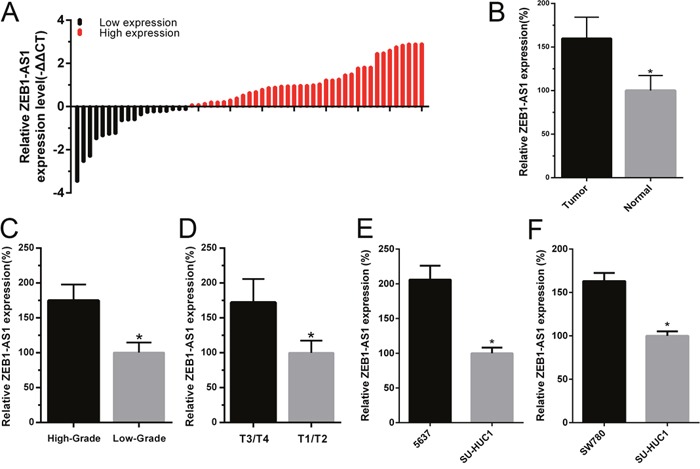
The relative expression of ZEB1-AS1 in bladder cancer tissues and cells The relative expression of ZEB1-AS1 was measured by qRT-PCR. The height of column represents the fold change (log2-transformed) in ZEB1-AS1 expression in a cohort of 55 patients with bladder cancer. **A**. ZEB1-AS1 expression was higher in bladder cancer tissues compared to paired noncancerous tissues **B**. ZEB1-AS1 expression was higher in patients with higher histological grade and advanced tumor stage T. **C** and **D**. Compared with SV-HUC-1, the ZEB1-AS1 expression was increased in bladder cancer cell 5637 **E**. and SW780 **F**. Data are shown as mean±SD (* p<0.05).

To investigate the role of lncRNA ZEB1-AS1 in bladder cancer, 55 pairs of bladder cancer tissue and paired noncancerous tissue samples were obtained to determine the expression of ZEB1-AS1 using qRT-PCR assay. The fold change of ZEB1-AS1 expression (bladder cancer tissue/paired noncancerous tissue) in each patient was shown in Figure 1A. Results also showed that the expression level of ZEB1-AS1 was significantly up-regulated in bladder cancer tissues compared to paired noncancerous tissues (Figure 1B). Moreover, the relative expression of ZEB1-AS1 was significantly higher in patients with higher histological grade (Figure 1C) and advanced tumor stage (Figure 1D). As shown in Table 1, the increased ZEB1-AS1 expression was positively correlated with higher histological grade and advanced tumor stage. However, gender, age, multiplicity, tumor size, lymph nodes metastasis and distal metastasis were not correlated with expression of ZEB-AS1. Likewise, the ZEB1-AS1 expression was elevated in bladder cancer cells 5637 and SW780 compared to the SV-HUC-1 cell line. The relative expression level of ZEB1-AS1 was 205.96±20.04% in 5637 cells and 162.99±9.47% in SW780 cells, respectively (Figure 1E and 1F). These data indicated that ZEB1-AS1 may act as an oncogene in bladder cancer. The statistical results and clinical parameters of 55 bladder cancer patients are listed in Table 1 and Table 2, respectively.

**Table 1:** Correlation between ZEB1-AS1 expression and clinicopathological features of UCB patients

Characteristics	Group	Total	ZEB1-AS1 expression		*P value*
			High	Low	
**Gender**	Male	40	26	14	0.792
	Female	15	11	4	
**Age (years)**	< 60	20	14	6	0.745
	≥ 60	35	23	12	
**Tumor size (cm)**	< 3 cm	21	14	7	0.940
	≥ 3 cm	34	23	11	
**Multiplicity**	Single	33	20	13	0.197
	Multiple	22	17	5	
**Histological grade**	Low	23	10	13	0.001*
	High	32	27	5	
**Tumor stage (T)**	T1,T2	38	22	16	0.027*
	T3,T4	17	15	2	
**Lymph nodes metastasis**	NO	53	35	18	1.000
	YES	2	2	0	

* P<0.05 was considered significant

**Table 2:** Summary of clinicopathological features of tissues of bladder cancer

Pt No.	Sex	Age	Stage	Grade
1	M	66	T2bN0M0	H
2	F	64	T1N0M0	L
3	F	38	T3aN0M0	H
4	M	75	T2bN0M0	H
5	M	58	T3aN0M0	H
6	M	65	T2bN0M0	H
7	M	53	T1N0M0	L
8	M	59	T2bN0M0	H
9	M	43	T3aN0M0	H
10	F	64	T2bN0M0	H
11	M	63	T2bN0M0	H
12	M	72	T3aN0M0	H
13	M	69	T1N0M0	L
14	M	68	T2bN0M0	H
15	F	63	T3aN0M0	H
16	F	89	T1N0M0	L
17	M	78	T2aN0M0	L
18	M	70	T2aN0M0	L
19	F	41	T2aN0M0	L
20	M	59	T2bN0M0	H
21	F	73	T2aN0M0	L
22	M	67	T2bN0M0	H
23	F	61	T3aN0M0	H
24	M	58	T4aN3M0	H
25	M	63	T2aN0M0	L
26	F	51	T1N0M0	L
27	M	86	T1N0M0	L
28	M	59	T4N0M0	H
29	F	62	T4aN0M0	H
30	M	58	T4aN0M0	H
31	M	50	T2bN0M0	H
32	M	54	T2bN0M0	H
33	M	63	T2aN0M0	L
34	M	41	T1N0M0	L
35	M	62	T4aN0M0	H
36	M	76	T2bN0M0	L
37	F	74	T3aN0M0	H
38	M	25	T1N0M0	L
39	F	70	T1N0M0	L
40	F	72	T1N0M0	L
41	M	73	T3bN0M0	H
42	M	63	T3aN0M0	H
43	M	46	T1N0M0	L
44	M	57	T4aN0M0	H
45	M	70	T2bN0M0	H
46	M	77	T3aN0M0	H
47	M	66	T1N0M0	L
48	M	53	T2aN0M0	L
49	M	49	T1N0M0	L
50	M	47	T2aN0M0	L
51	M	68	T1N0M0	L
52	M	73	T2bN1M0	H
53	F	74	T2bN0M0	H
54	F	60	T2aN0M0	H
55	M	61	T3aN0M0	H

### ZEB1-AS1 specific small interfering RNA suppress the expression of ZEB1-AS1 in bladder cancer cells

**Figure 2:**
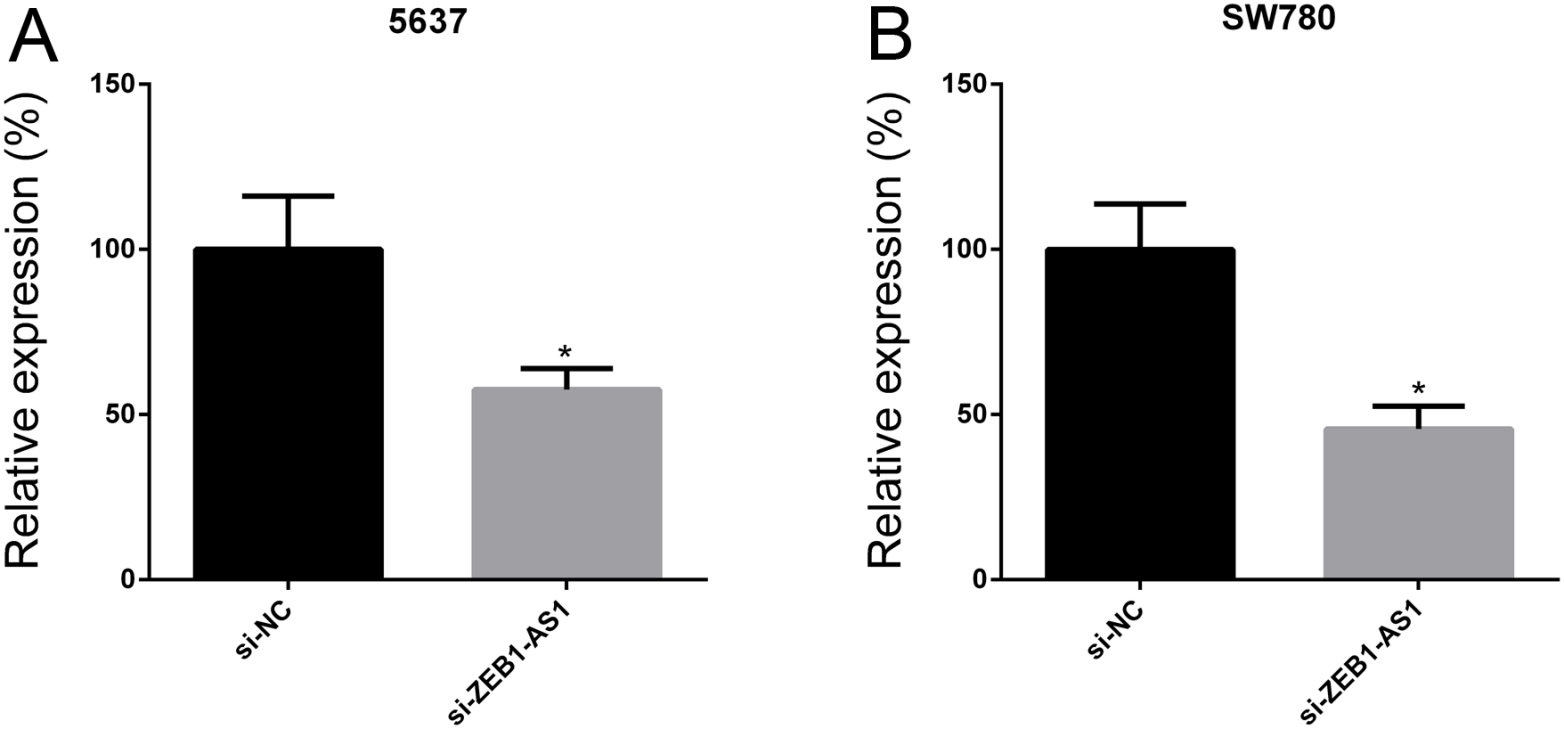
ZEB1-AS1 specific small interfering RNA suppressed ZEB1-AS1 expression in bladder cancer cells The relative expression of ZEB1-AS1 was decreased in si-ZEB1-AS1 group compared to si-NC group in bladder cancer cell 5637 **A**. and SW780 **B**. Data are shown as mean±SD (* p<0.05).

To investigate the function of ZEB1-AS1 in bladder cancer, we inhibited ZEB1-AS1 expression in bladder cancer cells 5637 and SW780 by transfecting ZEB1-AS1 specific siRNAs (si-ZEB1-AS1). Then we performed qRT-PCR to measure the expression of ZEB1-AS1 at 48h post-transfection. The data showed that si-ZEB1-AS1 could suppress the expression of ZEB1-AS1 in bladder cancer cells. The inhibitory rate (si-ZEB1-AS1/si-NC) was 57.53±6.37% in 5637 cells and 45.63±6.89% in SW780 cells, respectively (Figure 2A and 2B).

### Down-regulation of ZEB1-AS1 inhibits proliferation of bladder cancer cells

**Figure 3:**
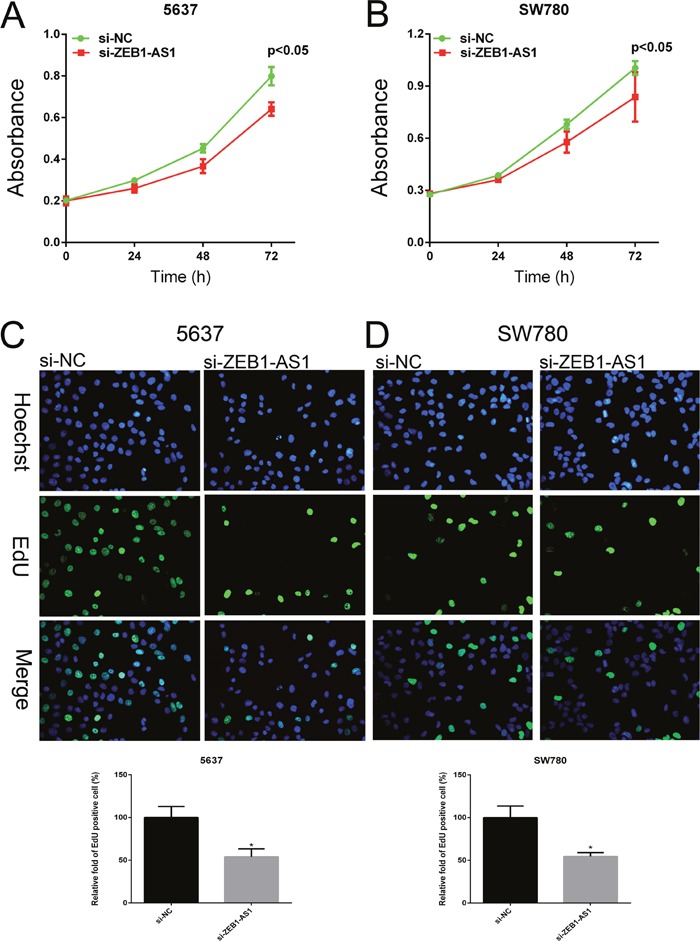
Down-regulation of ZEB1-AS1 inhibited proliferation of bladder cancer cells Growth of cell was suppressed in si-ZEB1-AS1 group compared to si-NC group in bladder cancer cell 5637 **A**. and SW780 **B**. by using CCK-8 assay. EdU positive cells were decreased in si-ZEB1-AS1 group compared to si-NC group in bladder cancer cell 5637 **C**. and SW780 **D**. by using EdU assay. Data are shown as mean±SD (* p<0.05).

We performed CCK-8 assay and Edu assay to observe the proliferation of bladder cancer cells at 48h post-transfection of si-ZEB1-AS1. Cell Counting Kit-8 assays showed that si-ZEB1-AS1 inhibits cell growth compared to the negative control in bladder cancer cells 5637 and SW780 (Figure 3A and 3B). Edu incorporation assays showed that Edu-positive cells decreased after ZEB1-AS1 knockdown in bladder cancer cells 5637 and SW780. These results demonstrated that down-regulation of ZEB1-AS1 could reduce cell proliferation compared to the negative control group (Figure 3C and 3D).

### Down-regulation of ZEB1-AS1 inhibits migration of bladder cancer cells

**Figure 4:**
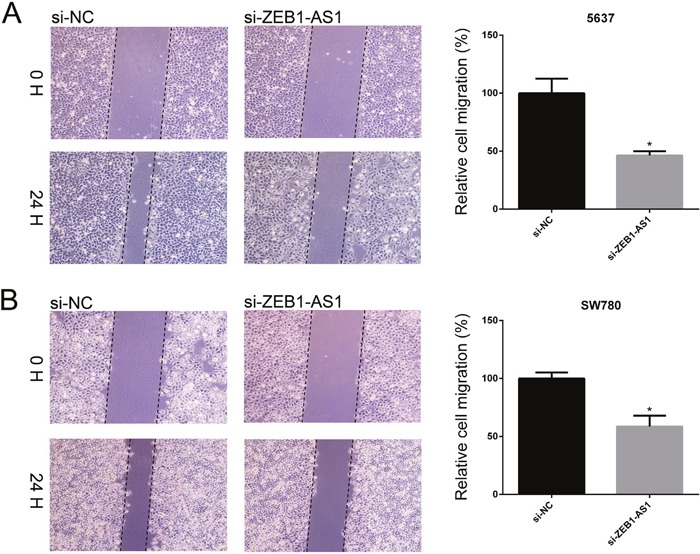
Down-regulation of ZEB1-AS1 inhibited migration of bladder cancer cells by wound-healing assay The rate of cell migration was decreased in si-ZEB1-AS1 group compared to si-NC group in bladder cancer cell 5637 **A**. and SW780 **B**. by using wound-healing assay. Data are shown as mean±SD (* p<0.05).

**Figure 5:**
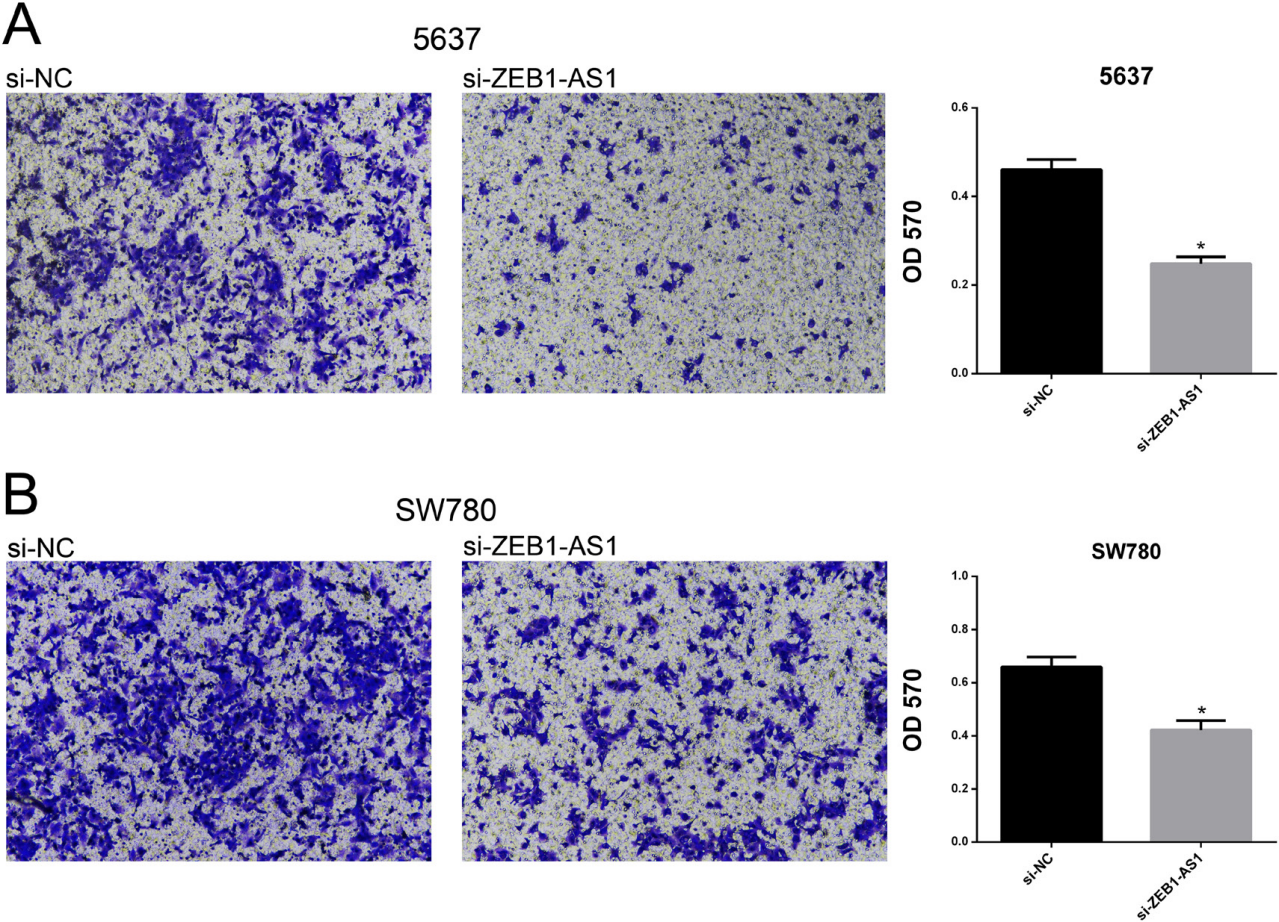
Down-regulation of ZEB1-AS1 inhibited migration of bladder cancer cells by transwell assay Motility of cells was suppressed in si-ZEB1-AS1 group compared to si-NC group in bladder cancer cell 5637 **A**. and SW780 **B**. by using transwell assay. Data are shown as mean±SD (*p<0.05).

We performed wound-healing assays and transwell assays to measure cell migration of bladder cancer cells 5637 and SW780 at 48h post-transfection of si-ZEB1-AS1 or si-NC. As shown in Figure 4 and Figure 5, down-regulation of ZEB1-AS1 could decrease cell motility in bladder cancer cells.

### Down-regulation of ZEB1-AS1 induces apoptosis of bladder cancer cells

**Figure 6:**
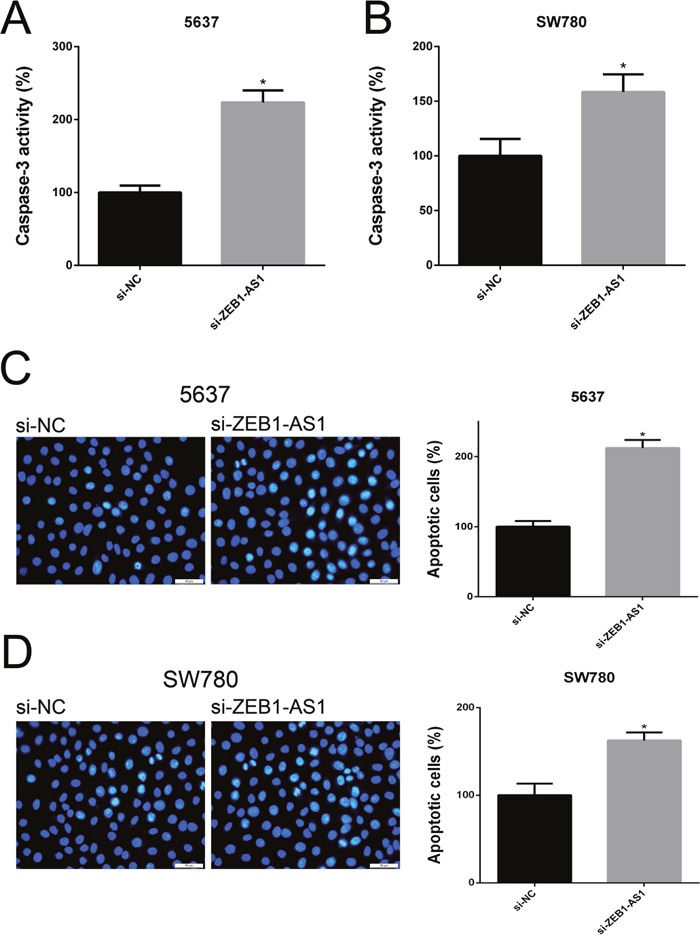
Down-regulation of ZEB1-AS1 induces apoptosis of bladder cancer cells by ELISA and Hoechst 33258 staining assay The relative activity of caspase-3 was increased in si-ZEB1-AS1 group compared to si-NC group in bladder cancer cell 5637 **A**. and SW780 **B**. by ELISA assay. More apoptosis cells were detected and the higher apoptosis ratio was measured in si-ZEB1-AS1 group compared to si-NC group in bladder cancer cell 5637 **C**. and SW780 **D**. by Hoechst 33258 staining assay. Data are shown as mean±SD (* p<0.05).

**Figure 7:**
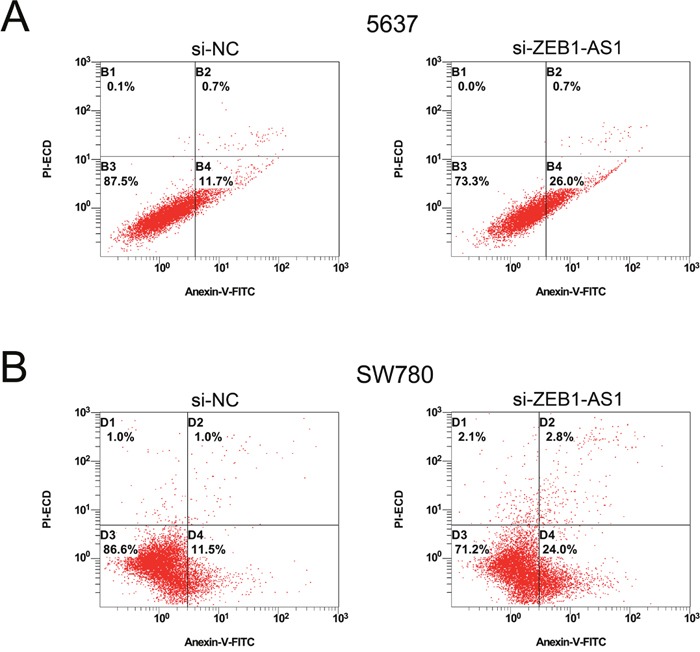
Down-regulation of ZEB1-AS1 induces apoptosis of bladder cancer cells by flow cytometry assay More early apoptotic cells were detected in si-ZEB1-AS1 group compared to si-NC group in bladder cancer cell 5637 (*P* < 0.05) **A**. and SW780 (*P* < 0.05) **B**.

We performed ELISA assay, Hoechst staining assay and flow cytometry assay to investigate the apoptosis of bladder cancer cells at 48 post-transfection of si-ZEB1-AS1 or si-NC. The results showed that the apoptosis ratio and the activity of caspase-3 were increased after ZEB1-AS1 knockdown in bladder cancer cells by Hoechst staining and ELISA assay, respectively (Figure 6). These findings were also confirmed by flow cytometry assay (Figure 7). All of these results demonstrated that down-regulation of ZEB1-AS1 could induce apoptosis of bladder cancer cells 5637 and SW780.

## DISCUSSION

Bladder cancer is one of the most common urinary malignancies worldwide. Most patients are diagnosed at advanced stage of bladder cancer because patients with bladder cancer are lack of specific symptoms at early stage. Although the treatments of bladder cancer include surgery, chemotherapy and immunotherapy, the prognosis remains quite poor in the patients with advanced stage. Thus, finding novel molecular targets for bladder cancer has enormous potential to improve the diagnosis and prognosis of bladder cancer.

LncRNAs are one member of ncRNAs and they are emerging as important players in carcinogenesis and progression [5]. Recent studies showed that specific lncRNAs are elevated or descending in different cancers and their expression is more specific than protein-coding genes’ [9–11]. They may provide us new biomarkers for diagnosis and prognosis of cancers [18]. ZEB1-AS1 is a non-coding antisense transcript generating from ZEB1 promoters. Emerging studies showed that overexpression of ZEB1-AS1 increased ZEB1 expression and acted as an oncogene in HCC cells [17]. However, the relation between ZEB1-AS1 and bladder cancer has not yet been investigated.

In this research, we detected the expression of lncRNA ZEB1-AS1 in bladder cancer tissues and paired noncancerous tissues. It showed that ZEB1-AS1 was up-regulated in bladder cancer tissues. Moreover, up-regulated ZEB1-AS1 expression was positively correlated with higher histological grade and advanced tumor stage T, both of which predicted a poor prognosis in bladder cancer [19–22]. These results suggest that lncRNA ZEB1-AS1 may become a new player in the state of bladder cancer. To reveal the biological functions of lncRNA ZEB1-AS1 in bladder cancer, we performed the cell proliferation, migration and apoptosis by silencing ZEB1-AS1 in the bladder cancer cells. Our loss-of-function experiments indicated that down-regulation of ZEB1-AS1 not only could suppress cell proliferation and migration but also could induce cell apoptosis in bladder cancer cell lines 5637 and SW780 by using CCK-8, Edu assays, wound-healing assays, transwell assays, ELISA assays, Hoechst staining and flow cytometry assays. These findings indicated that ZEB1-AS1 may play roles in the development of bladder cancer.

In conclusion, the expression of lncRNA ZEB1-AS1 is up-regulated in bladder cancer tissues compared with paired non-tumor tissues. High expression of ZEB1-AS1 is associated with higher histological grade and advanced tumor stage T in bladder cancer. It is likely due to the ability of ZEB1-AS1 to promote tumorigenesis and development in bladder cancer cells. Cumulatively, these results indicate that ZEB1-AS1 plays crucial roles in bladder cancer and ZEB1-AS1 may be a new molecular biomarker for the prognosis of bladder cancer and a novel target of new therapy.

## MATERIALS AND METHODS

### Cell lines and cell culture

Human bladder cancer cells (5637, SW780) and normal bladder epithelial cell (SV-HUC-1) that we used in this study were purchased from the Institute of Cell Research, Chinese Academy of Sciences, Shanghai, China. The 5637 cells and SW780 cells were grown in RPMI-1640 medium and DMEM medium supplemented with 1% antibiotics (100μg/ml streptomycin sulfates and 100U/ml penicillin) and 10% fetal bovine serum respectively. The SV-HUC-1 cells were grown in F-12K medium plus 1% antibiotics and 10% fetal bovine serum. All of the cells were placed at 37 °C in 5 % CO_2_.

### Tissues samples

A total of 55 bladder cancer tissues and paired adjacent normal tissues were isolated from the patients diagnosed with bladder cancer. The bladder cancer tissue samples were isolated from the main part of the bladder cancer. The paired normal tissue samples were isolated from the normal tissue of urinary bladder. It must be finished in 30 minute from the resection of bladder cancer to the tissue isolation. All the tissue samples were cleaned by cold physiological saline and stored in -20 °C with RNALater or stored in liquid nitrogen. It must be finished in 45 minute from tissues isolation to tissue preservation. All the bladder cancer tissues used for isolation were verified as urothelial neoplasia with standard pathomorphological evaluation. The necrotic tissues were excluded in the research. Written formal approval was also obtained from the patients. This study was approved by the Institutional Review Board of Shenzhen Second People's Hospital.

### Synthesis of small interfering RNA and transfection of cells

According to a previous study [23, 24], bladder cancer cells 5637 and SW780 were transfected with specific siRNA targeting ZEB1-AS1 (si-ZEB1-AS1). The sequences of siRNA were 5′-CCACAGGCCATGAATTCCTTCCTAA-3′. The si-ZEB1-AS1 and Non-specific siRNA (si-NC) were purchase from GenePharma, Suzhou, China. The untreated bladder cancer cells were cultured 24h before transfection. We treated cells with si-ZEB1-AS1 (100nM) and si-NC (100nM) complex with Lipofectamin 2000 Transfection Reagent (Invitrogen, Carlsbad, CA, USA) according to the manufacturer's instructions.

### RNA extraction and qRT-PCR

Total RNAs from tissues and treated cells were extraction with TRIzol reagent (Invitrogen, USA) according to the manufacturer's procedures. UV spectrophotometer analysis was performed to measure the purity and concentration of total RNAs. Synthesis of cDNA was generated by using SuperScript III^®^ (Invitrogen) following the manufacturer's instructions. After cDNA synthesis, it was primed to the MicroAmp Reaction Tubes with PCR reaction mix and specific primers of ZEB1-AS1. The primer sequences of ZEB1-AS1 were as follows: forward primer: 5′-CCGTGGGCACTGCTGAAT-3′, reverse primer: 5′-CTGCTGGCAAGCGGAACT-3′. Quantitative RT-PCR was carried out by utilizing a standard SYBR Green PCR Kit (Takara, Dalian, China) on the ABI PRISM 7000 Fluorescent Quantitative PCR System (Applied Biosystems, Foster City, CA, USA). In this Quantitative PCR System, PCR reaction was repeated 40 cycles and each cycle comprised Denaturation (95 °C for 5 sec), Annealing (55 °C for 30 sec) and Extension (72 °C for 30 sec). Expression of relative genes was analyzed by using 2−ΔΔCt methods. Experiments were repeated five times.

### Cell proliferation assay

Briefly, 5 × 10^3^ cells/well were inoculated into a 96-well plate and cultured for 24 h, then transfected with siRNA. Cell proliferation was assessed by using Cell Counting Kit-8, CCK-8 (Beyotime Institute of Biotechnology, Shanghai, China). Finally, absorbance was measured after 24-, 48- and 72 hours transfection by an ELISA microplate reader (Bio-Rad, Hercules, CA, USA). Ethynyl deoxyuridine (Edu) assay was performed by using an Edu Kit (Ribobio, Guangzhou, China) following the manufacturer's instructions. Experiments were repeated five times.

### Cell motility assay

Cell motility was assessed by wound-healing assay and transwell assay. For the wound-healing assay, a wound field was performed by a 200μl plastic pipette tip. The migration distance was observed after 24 hours of wound formation and measured by the software program HMIAS-2000. For the transwell assay, bladder cancer cells transfected with siRNAs were cultured in the upper chamber for 48 hours. At 48h after culture, the membranes were stained with crystal violet and the migration of cells was photographed under an inverted microscope and was measured by an ELISA microplate reader (Bio-Rad, Hercules, CA, USA). Experiments were repeated five times.

### Cell apoptosis assay

Cell apoptosis was detected by Hoechst 33258 staining assay, caspase-3 enzyme linked immunosorbent assay (ELISA) and flow cytometry assay. For Hoechst 33258 staining assay, the Hoechst 33258 staining kit (Beyotime, Shanghai, China) was used to observe the apoptotic cells and measure the apoptosis ratio of cancer cells at 48h post-transfection of si-ZEB1-AS1 or si-NC. For ELISA assay, a Caspase-3 Colorimetric Assay kit (Abcam, Cambridge, UK) was used to measure the relative activity of cleaved caspase-3 in cancer cells at 48h post-transfection. For flow cytometry assay, bladder cancer cells were collected at 48h post-transfection and double stain the cancer cells with FITC-Annexin V and PI by utilizing FITC Annexin V Apoptosis Detection Kit (TransGen, Perking, China) following the manufacturer's instructions. After double staining, a flow cytometer (EPICS, XL-4, Beckman, CA, USA) was used to measure the apoptosis of bladder cancer cells. In the graphs, cells were distinguished into four regions which represented dead cells, living cells, early apoptotic cells and late apoptotic cells, respectively. The percentage of region which represented early apoptotic cells in the graph was measured and subjected to quantitative comparison. Experiments were repeated five times.

### Statistical analyses

All data were analyzed from three independent experiments. Paired samples’ t-test, independent samples’ t-test or ANOVA was performed as indicated, and P < 0.05 was deemed statistically significant. All statistical tests were executed by SPSS version 19.0 software (SPSS Inc. Chicago, IL, USA).
